# Patient-derived organoids reveal marked heterogeneity in chemosensitivity profiles of colorectal cancer and a potential association with HER2 status

**DOI:** 10.3389/fphar.2025.1620764

**Published:** 2025-06-10

**Authors:** Jian Liu, Dan Li, Jing Li, Wenlan Fu, Qiang Jia, Yang Bai, Axiu Huang, Fang Chen

**Affiliations:** ^1^ Department of Clinical Laboratory, Second People’s Hospital of Jiangyou, Mianyang, China; ^2^ Department of Oncology, Second People’s Hospital of Jiangyou, Mianyang, China; ^3^ Chengdu Tianfu Organoid Biobank Co., LTD, Chengdu, China

**Keywords:** colorectal cancer, patient derived tumor organoids, chemotherapy, drug sensitivity, HER2 heterogeneity

## Abstract

This study aimed to evaluate the sensitivity and heterogeneity of standard first-line chemotherapy regimens for colorectal cancer (CRC) using patient-derived tumor organoids (PDTOs). Drug sensitivity testing in 15 CRC PDTOs revealed varying proportions of samples classified as sensitive (inhibition rate >50%) across different regimens: FOLFIRI (60%), FOLFOX (40%), CAPEOX (26.7%), and 5-Fluorouracil (26.7%). Furthermore, exploratory analysis indicated that for FOLFIRI and FOLFOX regimens, HER2(1+) status was significantly associated with lower inhibition grades compared to HER2(0) status (*P* < 0.05), implying a potential impact on the level of drug response. These findings demonstrate significant heterogeneity in the response of CRC PDTOs to first-line chemotherapies. Furthermore, at the organoid level, a low HER2 expression status may be associated with the heterogeneity of responses observed with specific drug regimens.

## Introduction

Clinical responses to standard chemotherapy regimens for CRC exhibit significant inter-patient variability (Fyfe, 2023; Picco et al., 2021), highlighting the lack of reliable predictive biomarkers to guide personalized therapy. PDTOs have emerged as advanced preclinical models capable of preserving key histopathological and genetic features of the original tumor (Booij et al., 2022; Veninga and Voest, 2021), thereby providing a valuable platform for assessing individual drug sensitivities. This study employed PDTOs established from 15 distinct CRC patients to meticulously profile their response landscape to a panel of standard-of-care chemotherapeutic drugs and combinations, illustrating the substantial inter-patient heterogeneity in chemosensitivity and the considerable potential of PDTOs as preclinical models for drug efficacy screening.

Human epidermal growth factor receptor 2 (HER2) is widely recognized as an effective target for targeted therapy in colorectal cancer (Suwaidan et al., 2022); however, its role in the context of conventional chemotherapy has received limited investigation. To date, no definitive reports have demonstrated that HER2 status significantly modulates chemotherapeutic outcomes at the population level. Nevertheless, at the cellular level, studies have indicated a discernible association between HER2 positivity (defined as immunohistochemistry [IHC] 3+, or IHC 2+ coupled with fluorescence *in situ* hybridization [FISH] amplification) and resistance to chemotherapeutic agents such as 5-fluorouracil (5-FU) (Long et al., 2022) and oxaliplatin (Pirpour Tazehkand et al., 2018). This suggests that the relationship between HER2 expression status and chemosensitivity in CRC is complex and context-dependent. To further elucidate the association between HER2 status and sensitivity to commonly used chemotherapeutic agents in CRC, we conducted an exploratory analysis using PDTOs, a model system whose biological characteristics are considered intermediate between *in vivo* patient tumors and conventional *in vitro* cell lines. This analysis examined potential correlations between observed drug sensitivity patterns and HER2 expression status (0 vs 1+), aiming to explore the multifaceted relationship between HER2 status and drug sensitivity.

## Materials and methods

### Colonic tumor organoids culture

Tumor samples for PDTOs culture were obtained from surgical resections of primary stage II or III CRC. Following acquisition, tumor tissue was isolated and digested using a digestive solution (MasterAim^®^). 50,000–60000 isolated cells mixed with cold Matrigel Basement Membrane Matrix (CORNING) and 50 µL drops of Matrigel-cell suspension were allowed to solidify on prewarmed 24-well suspension culture plates at 37°C for 30 min. Upon complete gelation, 500 µL of organoid medium was added to each well and plates were transferred to humidified 37°C/5% CO_2_ incubator. The pellet was resus pended with colonic tumor organoid culture medium (MasterAim®Colorectal Cancer Organoid Kit, 10–100–066). The culture was replenished with fresh media every 3–4 days during organoid growth. Dense cultures with organoids were usually passaged with a split ratio of 1:3 every 2–3 weeks by dissociation with TrypLE Express (Gibco) and re-seeded into new Matrigel.

### Organoid drug screening

10 µL of Matrigel was dispensed into 384-wellmicroplates and allowed to polymerize. Cells from organoid were plated (1.5–2 × 10^3^ per well) and cultured in 384-well culture plates (CORNING) for 48 h, and drugs were added to the culture medium at a final concentration. Test agents were primarily selected based on first-line chemotherapy regimens recommended by the Chinese Society of Clinical Oncology guidelines for CRC (2024), including: CAPEOX (Capecitabine + Oxaliplatin), FOLFOX (Calcium Folinate + 5-Fluorouracil + Oxaliplatin), FOLFIRI (Calcium Folinate + 5-Fluorouracil + Irinotecan), FOLFOX + Cetuximab, 5-Fluorouracil, and Cetuximab. The concentration for each individual drug component was: Oxaliplatin 1μM, 5-Fluorouracil 5μM, Irinotecan 5μM, Calcium Folinate 5μM, Capecitabine 5μM, and Cetuximab 1 μM. After 3 days of drugs incubation, cell viability was assayed using Cell Titer-Glo 3D Reagent (Promega) in accordance with the manufacturer’s instructions. 0.1% dimethyl sulfoxide was used as a control. When the ratio of the average level of cell viability in the presence of the drugs (n = 2) compared to the control (n = 2) was under 0.7, and the suppressive effect was considered to be significant. Inhibition rates were categorized into four grades for analysis: Grade A (≥70%), Grade B (50% to <70%), Grade C (30% to <50%), and Grade D (<30%). Drug inhibition rates were calculated using the formula: Inhibition Rate (%) = [1 - (Chemiluminescence_drug - Chemiluminescence_blank)/(Chemiluminescence_negative_control - Chemiluminescence_blank)] * 100%. Compared to the drug group, the negative control group received no drug, and the blank group contained no organoids; all other conditions were kept the same.

### Histological analysis and immunohistochemistry

Tumor tissue and organoids were fixed with 4% paraformaldehyde overnight, washed, and embedded into paraffin blocks. Sections (four to five µm) were deparaffinized andstained with hematoxylin and eosin (H&E) for histological analysis. For Immunohistochemistry, after sections were made and hydrated, they were incubated with blocking buffer with H_2_O_2_ for 15 min and boiled with citrate (pH = 6.0). After cooling down, sections were treated with pre-blocking buffer and incubated with primary antibodies at 4°C overnight. Sections were incubated with secondary antibodies and HRP (horse radish peroxidase). Primary antibodies were used including CK7 (HUA Bio, ET1609-62), CK20 (HUA Bio, ET1601-8) and Epcam (CST, 2929 S).

Descriptive statistics were employed to summarize sample characteristics, clinical information, and inhibition rate results across different drug regimens. Associations between inhibition rate grades and HER2 status derived from immunohistochemistry were evaluated using the Mann-Whitney U test.

## Results

**FIGURE 1:**
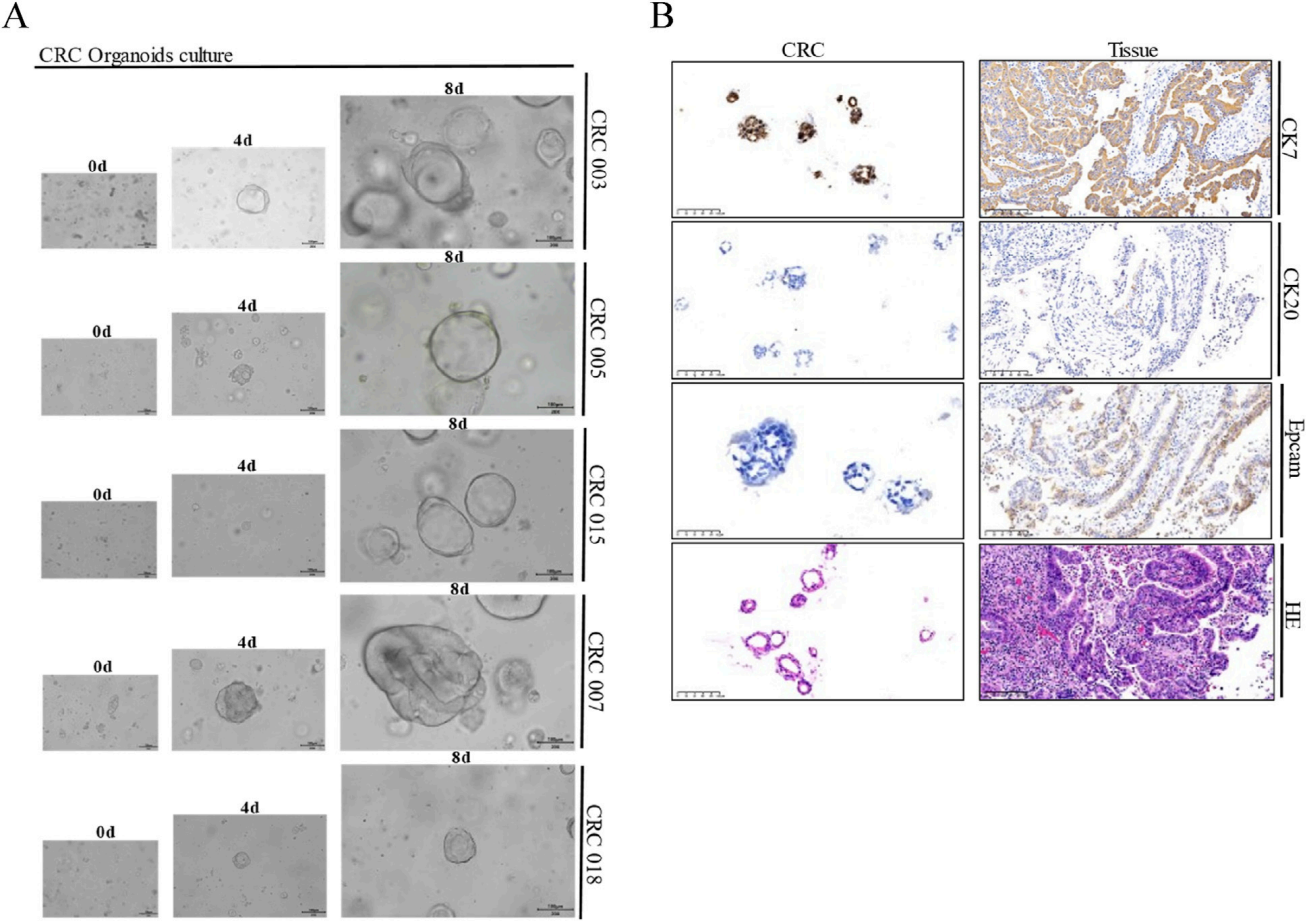
Representative morphology and comparative immunohistochemistry of PDTOs and corresponding parental tissues. **(A)** Representative bright field images of CRC tumor organoids from seven different patients. CRC organoids tends to more shaped thin-walled cystic structures. Case ID was named according to experimental specimen number. Scale bar: 100 μm; **(B)** Representative H&E and immunohistochemistry staining of intestine tumor and derived organoid lines. Tissues generally present tumor epithelium surrounded by mes enchymal and inflammatory cells, while organoids are exclusively epithelial with tumor cell organization being remarkably well conserved. Scale bar:100 µm.

A total of 16 CRC tumor tissue samples were obtained, from which 15 PDTOs were successfully established. Subsequent immunohistochemistry analysis confirmed good concordance between these organoids and their corresponding parental tissues (Figure 1). The cohort comprised six male and nine female patients, with an age range of 51–80 years. Tumor staging revealed 7 cases of stage II and 8 cases of stage III adenocarcinoma. Immunohistochemistry indicated proficient mismatch repair (pMMR) status in all samples, with expression of MLH1 (+), MSH2(+), MSH6(+), and PMS2(+). HER2 status was negative (score 0) in 10 cases and weakly positive (score 1+) in 5 cases.

**FIGURE 2:**
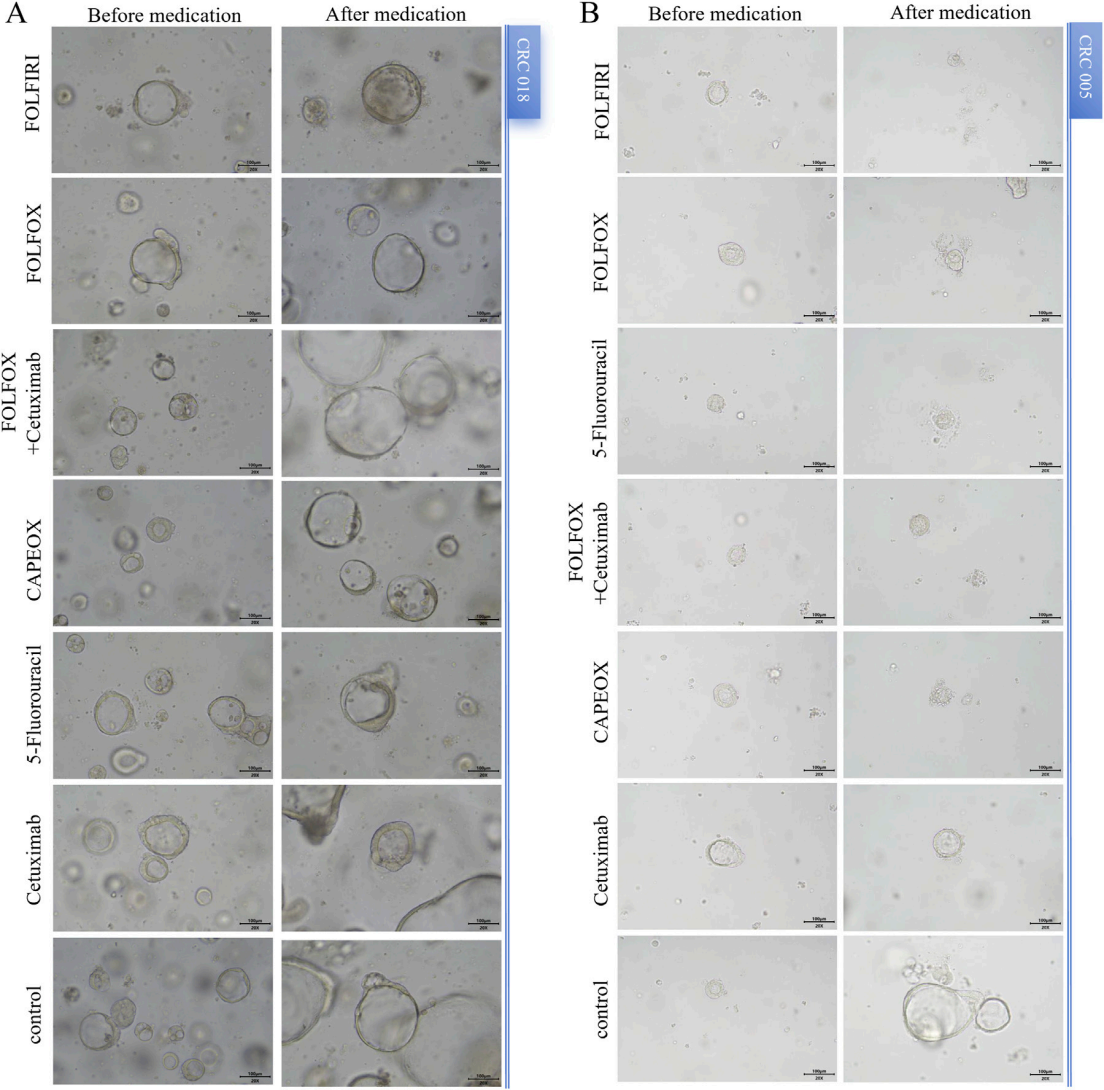
Morphological changes in PDOTs before and after drug treatment. **(A)** and **(B)** Representative images of PDTOs treated with different tumor drugs on day 3 of the assay. Case ID was named according to Experimental Specimen Number. Scale bar: 100 µm.

**FIGURE 3:**
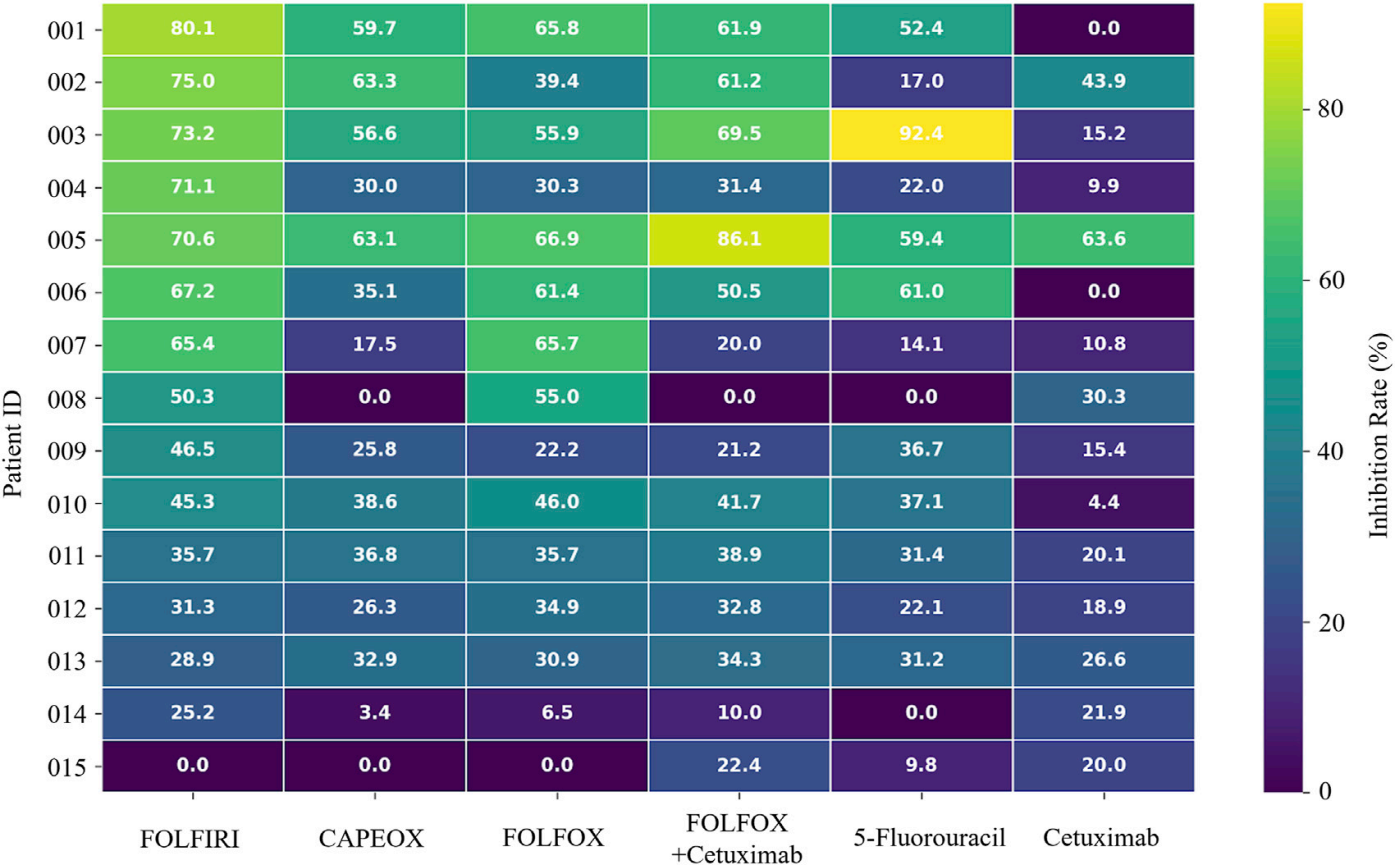
Heatmap of drug inhibition rates (%).

The organoids exhibited significant morphological changes following drug treatment (Figure 2). Significant heterogeneity was observed in the inhibition rates of the different PDTOs in response to the tested drugs (Figure 3; Table 1). The drug regimen with the highest mean inhibition rate was FOLFIRI (mean ≈51%, SD ≈ 23%), followed by FOLFOX (mean ≈41%, SD ≈ 21%). The lowest mean inhibition was observed with Cetuximab monotherapy (mean ≈20%, SD ≈ 17%). For the majority of patients, the inhibition rates of combination therapy were higher than those of monotherapy. However, in one sample, the inhibition rate for 5-Fluorouracil monotherapy was substantially higher than that observed with combination therapies.

**TABLE 1:** The rates (%) of inhibition grade corresponding to different drugs.

Test drug	A (%)	B (%)	C (%)	D (%)
5-Fluorouracil	6.7	20	26.7	46.7
FOLFIRI^a^	33.3	26.7	20	20
CAPEOX	0	26.7	26.7	46.7
FOLFOX	0	40	40	20
Cetuximab	0	6.7	13.3	80
FOLFOX + Cetuximab	6.7	26.7	33.3	33.3

^a^ For the FOLFIRI, regimen, the percentages of subjects achieving inhibition rates of Grade A, B, C, and D were 33.3%, 26.7%, 20%, and 20%, respectively. Furthermore, the percentage of subjects achieving Grades A and B combined was the highest among all regimens, at 60%.

Stratified analysis revealed that for the FOLFOX, FOLFIRI, and FOLFOX + Cetuximab combination regimens, HER2(1+) status was significantly associated with lower inhibition grades (indicating poorer drug efficacy) compared to HER2(0) status (*P* < 0.05). Conversely, for CAPEOX, single-agent 5-Fluorouracil, and single-agent Cetuximab, although a trend towards poorer efficacy in the HER2(1+) group was observed, this difference did not reach statistical significance within our study’s sample size (*P* > 0.05). Detailed data are presented in Table 2.

**TABLE 2:** Stratified analysis of inhibition grade by HER2 status for different drug regimens.

Statistical indicators	5-Fluorouracil	FOLFIRI	CAPEOX	FOLFOX	Cetuximab	FOLFOX+ Cetuximab
U	17.0	7.5	11.5	2.5	16.0	7.0
*P* ^a^	0.265	0.029	0.080	0.004	0.152	0.024

^a^ Statistical significance was defined as *P* < 0.05.

## Discussion

This study successfully established PDTOs from stage II and III colorectal cancer patients, demonstrating good concordance with parental tissues. These PDTO models clearly illustrated significant heterogeneity in sensitivity among CRC patients to standard first-line chemotherapy, supporting the potential of PDTOs as a preclinical platform for capturing individual variations and exploring personalized therapeutic strategies. Notably, although FOLFIRI exhibited the highest *in vitro* response rate (60% Grade A + B) in our PDTOs, this contrasts with current guidelines generally not recommending its use in the adjuvant setting for stage II/III CRC (Harada and Sakamoto, 2022), a discrepancy also noted in other studies (National Health Commission of the People’s Republic of China, 2023; Ganesh et al., 2019). This discrepancy may reflect the complex relationship between *in vitro* chemosensitivity and considerations of long-term *in vivo* efficacy and toxicity, underscoring the need for caution when translating PDTO findings to guide clinical decisions.

HER2-positive CRC is often more aggressive and associated with a relatively poorer prognosis (Chen et al., 2022; Wang et al., 2019). Chemotherapeutic agents such as 5-FU primarily kill tumor cells by inducing DNA damage and apoptosis. However, if HER2 is overactive, these survival pathways may be persistently activated, potentially attenuating chemotherapy-induced apoptosis. A study by Sirui Long et al. found that upregulation of certain HER2-related factors (e.g., ECM1) can activate the PI3K/Akt pathway, thereby conferring resistance to 5-FU in CRC cells (Long et al., 2022). Similarly direct evidence comes from oxaliplatin-resistant colon cancer cell lines established by Pirpour Tazehkand et al., in which significantly elevated HER2 expression levels were found. The half-maximal inhibitory concentration (IC50) in these resistant cells increased 7–25-fold compared to parental cells. Furthermore, treatment of resistant cells with a HER2 inhibitor, leading to HER2 downregulation, significantly restored sensitivity to oxaliplatin and increased chemotherapy-induced cytotoxicity and apoptosis rates (Pirpour Tazehkand et al., 2018). In this study, at the organoid level, we revealed an association between low HER2 expression status (1+ vs. 0) and response grades for FOLFIRI and FOLFOX regimens (P < 0.05). This finding provides some support for the notion that HER2 overexpression can reduce sensitivity to certain chemotherapeutic agents by promoting survival signaling and altering cellular phenotype.

However, at the population level, multiple studies have not observed HER2 status to significantly alter chemotherapy efficacy or significantly impact patient survival outcomes after receiving chemotherapy (Jang et al., 2023; Richman et al., 2016). The observed disparity between findings at the organoid/cellular level and the patient level is likely multifactorial. Clinically, true HER2 amplification is rare (∼2–5%) and spatially heterogeneous; large adjuvant and metastatic trials therefore contained too few HER2-positive cases to detect small differences in response to standard fluoropyrimidine-, oxaliplatin- or irinotecan-based regimens (Richman et al., 2016; Battaglin et al., 2024). *In vitro*, oxaliplatin- or 5-FU-selected single-clone CRC cell lines carry very high HER2 copy numbers and face constant drug exposure, so the HER2-Akt/Nrf2 survival axis alone is sufficient to block apoptosis-conditions that do not recapitulate the fluctuating drug levels, immune attack and stromal barriers present in patients (Pirpour Tazehkand et al., 2018; Jang et al., 2023). Finally, tumour-derived CAF exosomes carrying miR-92a-3p and other effectors induce cross-resistance to 5-FU and oxaliplatin in both HER2-negative and -positive cells, biologically masking any HER2-specific contribution at the population level (Hu et al., 2019).

More prospective studies (e.g., stratified analysis of chemotherapy efficacy based on HER2 status), as well as in-depth molecular mechanistic studies, are warranted to fully elucidate the role of HER2 in CRC chemotherapy sensitivity and optimize individualized treatment strategies.

## Conclusion

This study provides a detailed view of the chemosensitivity landscape in a cohort of CRC PDTOs, demonstrating their potential as a preclinical drug screening model. Furthermore, this study suggests that HER2 status is a factor influencing the sensitivity of colorectal cancer to conventional chemotherapeutic agents, but may not be a strong determinant.

## Data Availability

The original contributions presented in the study are included in the article/supplementary material, further inquiries can be directed to the corresponding author.

## References

[B1] Battaglin F. Ou F. S. Qu X. Hochster H. S. Niedzwiecki D. Goldberg R. M. (2024). *HER2* gene expression levels are predictive and prognostic in patients with metastatic colorectal cancer enrolled in CALGB/SWOG 80405. J. Clin. Oncol. 42 (16), 1890–1902. 10.1200/JCO.23.01507 38457761 PMC11240881

[B2] Booij T. H. Cattaneo C. M. Hirt C. K. (2022). Tumor organoids as a research tool: how to exploit them. Cells 11 (21), 3440. 10.3390/cells11213440 36359838 PMC9653788

[B3] Chen N. Li C. L. Peng Y. F. Yao Y. F. (2022). Long-term follow-up of HER2 overexpression in patients with rectal cancer after preoperative radiotherapy: a prospective cohort study. World J. Gastrointest. Oncol. 14 (10), 2048–2060. 10.4251/wjgo.v14.i10.2048 36310698 PMC9611427

[B4] Fyfe I. (2023). Mutations linked to chemotherapy resistance in colorectal cancer. Nat. Rev. Gastroenterol. Hepatol. 20 (5), 269. 10.1038/s41575-023-00772-5 37012321

[B5] Ganesh K. Wu C. O'Rourke K. P. Szeglin B. C. Zheng Y. Sauvé C. E. G. (2019). A rectal cancer organoid platform to study individual responses to chemoradiation. Nat. Med. 25 (10), 1607–1614. 10.1038/s41591-019-0584-2 31591597 PMC7385919

[B6] Harada K. Sakamoto N. (2022). Cancer organoid applications to investigate chemotherapy resistance. Front. Mol. Biosci. 9, 1067207. 10.3389/fmolb.2022.1067207 36582205 PMC9792487

[B7] Hu J. L. Wang W. Lan X. L. Zeng Z. C. Liang Y. S. Yan Y. R. (2019). CAFs secreted exosomes promote metastasis and chemotherapy resistance by enhancing cell stemness and epithelial-mesenchymal transition in colorectal cancer. Mol. Cancer 18 (1), 91. 10.1186/s12943-019-1019-x 31064356 PMC6503554

[B8] Jang J. Y. Jeon Y. K. Jeong S. Y. Lim S. H. Park Y. S. Lim H. Y. (2023). Effect of human epidermal growth factor receptor 2 overexpression in metastatic colorectal cancer on standard chemotherapy outcomes. J. Gastrointest. Oncol. 14 (5), 2097–2110. 10.21037/jgo-23-375 37969818 PMC10643596

[B9] Long S. Wang J. Weng F. Pei Z. Zhou S. Sun G. (2022). ECM1 regulates the resistance of colorectal cancer to 5-FU treatment by modulating apoptotic cell death and epithelial-mesenchymal transition induction. Front. Pharmacol. 13, 1005915. 10.3389/fphar.2022.1005915 36408224 PMC9666402

[B10] National Health Commission of the People's Republic of China (2023). Chinese protocol of diagnosis and treatment of colorectal cancer (2023 edition). Chin. J. Dig. Surg. 22 (6), 667–698. 10.3760/cma.j.cn115610-20230526-00236

[B11] Picco G. Cattaneo C. M. van Vliet E. J. Crisafulli G. Rospo G. Consonni S. (2021). Werner helicase is a synthetic-lethal vulnerability in mismatch repair-deficient colorectal cancer refractory to targeted therapies, chemotherapy, and immunotherapy. Cancer Discov. 11 (8), 1923–1937. 10.1158/2159-8290.CD-20-1508 33837064

[B12] Pirpour Tazehkand A. Akbarzadeh M. Velaie K. Sadeghi M. R. Samadi N. (2018). The role of Her2-Nrf2 axis in induction of oxaliplatin resistance in colon cancer cells. Biomed. Pharmacother. 103, 755–766. 10.1016/j.biopha.2018.04.105 29684854

[B13] Richman S. D. Southward K. Chambers P. Cross D. Barrett J. Hemmings G. (2016). *HER2* overexpression and amplification as a potential therapeutic target in colorectal cancer: analysis of 3256 patients enrolled in the QUASAR, FOCUS and PICCOLO colorectal cancer trials. J. Pathol. 238 (4), 562–570. 10.1002/path.4679 26690310 PMC4785607

[B14] Suwaidan A. A. Lau D. K. Chau I. (2022). HER2 targeted therapy in colorectal cancer: new horizons. Cancer Treat. Rev. 105, 102363. 10.1016/j.ctrv.2022.102363 35228040

[B15] Veninga V. Voest E. E. (2021). Tumor organoids: opportunities and challenges to guide precision medicine. Cancer Cell. 39 (9), 1190–1201. 10.1016/j.ccell.2021.07.020 34416168

[B16] Wang X. Y. Zheng Z. X. Sun Y. Bai Y. H. Shi Y. F. Zhou L. X. (2019). Significance of HER2 protein expression and HER2 gene amplification in colorectal adenocarcinomas. World J. Gastrointest. Oncol. 11 (4), 335–347. 10.4251/wjgo.v11.i4.335 31040898 PMC6475672

